# ICT Solutions in the D-Sys-Com Research: Analysis of the Needs and Attitudes of the Frail Elderly Person

**DOI:** 10.3389/frobt.2022.851473

**Published:** 2022-05-25

**Authors:** Stefania Pinnelli

**Affiliations:** Centre for New Technologies for Inclusion and Disabilities, Department of History, Society, and Human Studies of the University of Salento, Lecce, Italy

**Keywords:** frail elderly people, telemedicine, quality of life, inclusion, universal design

## Abstract

The introduction and use of innovative technological devices to support the aging of frail elderly people does not necessarily correspond to an improvement in people’s quality of life. The strong technical curvature resulting from the use of telemedicine models often highlights limits in the usability of technologies in responding to the real needs of users. The theoretical framework of special pedagogy allows the assumption of the bio-psycho-social perspective and the constructs of quality of life and participation and opens up to inclusive logics that implement a profound and questioning reflection on all contexts of life, with the goal of exposing the set of disabling processes and indicating a valid support in the use of technological resources. The study, retracing the research phases of the Data System Platform for Smart Communities project (project admitted for funding in the Innolabs 2018–2019 call), completed in 2020, investigates the needs of strategic stakeholders and explores the factors that influence the adoption and diffusion of telemedicine devices by frail elderly people.

## 1 Introduction

The current paradigm of telemedicine oscillates between hyper-technicization and attention to the humanization instances of the patient and their caregivers, trying to meet the challenge of integrating relational *to care* into medical and welfare *to cure*. The critical reflection of this model refers to a series of multidisciplinary studies (Godfrey and Johnson, 2008) that can broaden the work perspective: in fact, the idea that the introduction and use of high-level technological devices necessarily correspond to an improvement in people’s quality of life shows enormous limitations. The presented case of study was carried out as part of the project D-Sys-Com—a project admitted for funding in the Innolabs 2018–2019 call—in Putignano (BA), with specific reference to the Municipality of Noci, in the south of Italy. The study provided an opportunity for a wide reflection on the risks of excessive technical curvature resulting from the use of telemedicine models. The contribution retraces the research phases of the *Data System Platform for Smart Communities* project, a Smart Social domain,^1^ completed by the CNTHI “Centre for New Technologies for inclusion and disabilities” (Department of History, Society, and Human Studies of the University of Salento). The main objective of the project was the creation of a technological dashboard aimed at increasing the effectiveness and efficiency of the communication between public institutions, private structures, and end users (patient and caregiver). The challenge has included the creation of a system of aggregation and intelligent use of heterogeneous data, coming from different sources and centralized in Cloud mode in relation to the specific needs of the single territory, and this proposal could reduce the social and health vulnerability of end users, in a context of high asymmetries and inequalities in the provision of social welfare and health services.

For this purpose, the project has adopted a participatory design model in order to highlight the specific characteristics with respect to local contexts, the criticalities and strengths of the network of social assistance services, and the specific needs of users.

The first phase of analysis identified the concrete needs of the stakeholders involved, highlighting the elements of resistance to the introduction of telemedicine at an organizational, cultural, and relational level by operators and users and the awareness of their know-how by public entities and private operators.

This allowed the CNTHI researchers to build the next phase of analysis whose purpose was to explore the factors that influence the adoption and diffusion of telemedicine devices by frail elderly people. Through the preparation of a questionnaire proposed to a sample of elderly people involved in the test of hardware and software technologies envisaged by the project, the attitudes, expectations, degree of satisfaction, and trust toward telemedicine services were investigated. The research was particularly complex, both for the interdisciplinary nature of the project and for the lack of knowledge in the use of participatory design techniques in the creation of telemedicine-assistance products.

## 2 The D-Sys-Com Platform for Smart Social: Materials and Methods

The objective of institutionalizing *initiative medicine*, which aims at shortening diagnostic and therapeutic times by allowing operators to quickly have the entire health framework of each patient and a greater possibility of consultation between colleagues, has required the involvement of public and private entities and professional figures not limited to the exclusive medical-health relevance.

*Initiative medicine* is a welfare model considered suitable to manage chronic diseases (diabetes, arterial hypertension, heart dysfunction ... ), constantly increasing as a result of the growing level of senility in the population (World Health Organization Europe, 2006).

In relation to the available literature, such an innovative paradigm necessarily requires the task of a therapeutic education of patients and families (Bodenheimer, Wagner, Grumbach, 2002). The greater the needs for assistance, the greater the effort made in helping people to become “experts” in their own health and in managing their own pathologies should be. Smart communities that adopt this approach activate processes built on “proactive” forms of territorial aggregation that take care of people with chronic diseases in an integrated way together with other appropriately trained professional figures (Wagner, 2000).

Systematic reviews of studies that have investigated the relationship between telemedicine tools and the quality of health and life of the elderly or ill people attest to very heterogeneous and sometimes conflicting results.

In a recent systematic review (Marzorati, et al., 2018), only one-third of the 24 studies considered used eHealth systems to study psychosocial variables such as perceived social well-being.

In most projects, telemedicine tools were considered a given: most studies focused on the effectiveness of the tool to promote caregiver well-being, overshadowing the usability and feasibility of eHealth programmes. The accessibility and usability of the technology have not often been evaluated, and still few studies have assessed user satisfaction or usage patterns of Web-based programmes.

Along with psychosocial variables, some studies have tracked user engagement and satisfaction with Web-based platforms or telemedicine interventions. All studies reported significant improvements in some of the areas studied, but often showed small effect sizes (ibid).

Since 2005, according to McCreadie and Tinker (2005), the interactions between a “felt need” for assistance, the recognition of “product quality,”the efficiency, reliability, simplicity, and safety of the technology, and its availability and cost, are the basic components of a complex model of acceptability for assistive technology to older people. Similarly, Melenhorst et al. (2001) suggested that the lack of perceived advantages, or benefits, might explain older adults’ reluctance to use digital technologies.

Unfortunately, there are only a few studies that have assessed correlations or mediated effects between different constructs of quality of life, depression, and self-efficacy.

The lack of investigation in this area may prevent a proper evaluation and improvement of the effectiveness of telemedicine tools implemented. In fact, without evaluating all aspects included in the concept of effectiveness (user satisfaction, usefulness, quality of interaction, and ease of use), it would be more difficult to understand if it is the specific tool that is not working or if it could even be telemedicine interventions in general (ibid).

Even in the study of the relationship between assistive technologies and the frail elderly person, biophysical and psychosocial factors have been neglected, although there is recognition of the importance of considering them in understanding how the elderly successfully interact with technological devices and systems (Chen and Chan, 2011) and it has been shown how patient involvement is critical to the success of a wound-monitoring protocol (Wiseman, et al., 2015).

The investigation that was conducted within the D-Sys-Com project sought to explore these issues.

The macro-objective of the Data System Platform for Smart Communities project Smart Social D-Sys-Com domain was the creation of a technological dashboard of telemedicine and welfare service, aimed at increasing the effectiveness and efficiency of the communication among public entities, private facilities, and end users (patients and caregivers). The challenge is to reduce social and welfare vulnerability of the end users in a context of high asymmetries and inequalities in the provision of social care and welfare services, through the creation of a system of aggregation and intelligent use of heterogeneous data, coming from different sources and centralized in Cloud mode in relation to the specific needs of a single territory. The D-Sys-Com platform should offer the organization of some chronic diseases based on multi-professional teams; implement initiative medicine projects on relevant chronic diseases (diabetes, cardiovascular risk, heart failure, and COPD); represent an information system to support the activities of health care and organization and intelligent use of data; and trigger a virtuous process of involvement of community resources.

As a result of these considerations, a research plan has been drawn up to provide an initial general picture of the situation to understand the most important characteristics of the phenomenon, thus developing two lines of analysis: a quantitative structural line, in which the socio-demographic and economic conditions of the target population emerge, and a qualitative line, which deepens the cultural, cognitive, and behavioral aspects of the users.

To this end, the following paragraphs will concisely present the results of the surveys and analyses carried out so far. First of all, however, a brief examination of the reflection about the application of digital hardware and software technologies in the medical and welfare field is necessary.

## 3 D-Sys-Com Research and Participatory Design Phases

Participatory design is an approach that aims at actively involving all stakeholders (employees, partners, customers, citizens, and end users) in the design process to ensure that the product meets real needs and is usable.

At a global level, a legislative discipline has been established in the field of participation, especially in the digital field; the main standards produced by the ISO *Technical Committee* 159/SC4, which highlight the importance of a user-centered design (Kensing, 2003), are well known.

Consideration of human factors in the design of interactive systems enhances their effectiveness and efficiency, improves human working conditions, and counteracts possible adverse effects of their use on health, safety, and performance because capacity, limitations, and the various human needs are taken into account. Participatory design is a design technique that gives a very important role to the contribution of end users in defining the utility and functionality that the system can have and does not only contribute to a better definition of effectiveness of systems but also to making users more aware of the processes that will take place, as they will be central actors in the executive phases. The involvement of users is essential to collect qualitative and quantitative data on the effectiveness of the product and to ensure a design for all (Rapp. 2014).

User-centered participation starts from an essential premise: knowledge of the target, knowledge of the context, and definition of the project development phase.

Phase one: a desk analysis was carried out on the socio-demographic characteristics of the population on the welfare characteristics of the territory in question and, as a second step, a subsequent focus group with some strategic stakeholders within the supply chain of welfare and relational care.

Phase two: Analysis of real users through a specific questionnaire and definition of the adaptations of technological experimentation.

Graph G.1 shows the analysis work of the CNTHI researchers in order to reach the subsequent involvement of the end users in the experimentation and testing of telemedicine models within the D-Sys-Com project.

## 4 Materials and Methods

### 4.1 Active Aging and Contextual Conditions in the Regional Health System of Puglia

Intervening in the context of an aging population means dealing with chronic diseases affecting elderly people. Chronical disorders are not curable, but a wise management of these diseases (disease management) can have an extremely significant impact on the quality of life of a person and their caregivers, extending their life expectancy.

Coronary heart diseases, osteoporosis, COPD (chronic pulmonary obstruction), cognitive impairment, and mellitus diabetes are the most common chronic diseases that are increasing considerably due to the growing senility of the population (World Health Organization Europe, 2020).

According to the available literature, disease management is based both on the set of the correct health interventions toward patients and on the educational processes aimed at elderly people and their families so that they can reach a good level of self-management of the disease with positive results (Cesarielli et al., 2017). The elderly person is therefore encouraged and supported to take an active role in the management of their pathology. All this requires a real transformation of the paradigm, from a waiting medicine to an initiative medicine, the latter made possible by a cooperative approach by collaborative models between professionals and families and by a prompt sharing of relevant clinical information. However, it should be stressed that, while, on one hand, medical knowledge and technology implementation have undergone a sharp upsurge over the past decade as they have progressed rapidly, on the other hand, it should be noted that scientific and technological innovation does not always result in an adequate application of technologies to support aging and chronic disease. There are many reasons for this. First of all, healthcare professionals, not only doctors but also pedagogists, psychologists, and caregivers, often rely on a partial clinical picture of the person, due to the fact that nowadays we have digital infrastructures that are not rewarded or updated in a timely manner. In support of this thesis, there are a whole series of experimental observations on the use of Edotto (the Health Information System of the Puglia Region, southern Italy) and on the bad assimilation of the regional computer system in which they have to upload the therapeutic plans and implement the electronic health files. Many IT services are poorly integrated and fragmented and people-related information is not shared, but it is often dispersed in heterogeneous archives. This has a major impact on the management of chronic diseases affecting the elderly population, which, on the contrary, require frequent monitoring.

The D-Sys-Com platform, Smart Social domain, aims to provide concrete answers to the needs expressed by end users, through an integrated visualization of medical and welfare data in real time in order to increase administrative efficiency between structures and provide immediate and consistent information to citizens. A “smart community” must be able to manage in an integrated way all the available information, so as to re-elaborate and redistribute them on the territory (Noci–Area of Putignano) converted into quality social-health and welfare services and in a better governance of the territory itself.

The creation of a strong involvement among all the stakeholders of the project through the creation of a shared system of knowledge is a key step toward understanding that technological support can have positive effects on the well-being and quality of life of the elderly people. This approach describes an active health system, the aim of which is to optimize the health status of the person, in contrast to many existing reactive systems, in which treatments are undertaken only when the patient manifests obvious symptoms that lead them to consult their doctor.

New technologies have a profound impact on communication processes and on structuring new settings, not only training settings but also care ones (Pinnelli et al., 2015). They are not just tools or devices, but real languages, which is why self-management of the pathology becomes a fundamental criterion. The patient must be able to share physiological parameters with the medical staff to access general and specific knowledge or simply to possess that basic digital literacy. As Pinnelli suggests (Pinnelli 2015, p. 174), computer science has a much faster pace of evolution than other domains of knowledge or compared to the consolidation of good practices in the health and welfare field. This highlights the risk, from care professionals or designers, to chase technological solutions free from that pedagogical and humanistic curvature able to qualify the cure as autonomy, independence, and quality of life. These aspects represent the trajectories of inclusive action toward the fragile elderly people, who should not be forced to accept a device by changing their behavior; on the contrary, they should receive a technology to meet their needs effectively and efficiently.

Based on this need to make ICTs (Information and Communication Technologies) valuable allies to support elderly people’s well-being and quality of life, in the European scenario, Ambient Assisted Living (AAL) supports the concrete needs of the person in their aging process by actively collaborating to achieve an independent and autonomous life (De Munari, Matrella and Ciampolini, 2013).

In this regard, WHO has provided a strategic framework, known as Active Aging, which considers the elderly person as an individual with rights, freeing us from that conception of the elderly person as a subject necessarily in need of help, against which the focus is on their deficit and pathology. WHO, thanks to ICF and the bio-psycho-social model, proposes work and intervention trajectories to support a better quality of life, which pay attention to all contexts of the life and development of the person with a particular disadvantage. In this context, technologies can intervene with a leading role in different areas of personal functioning.

### 4.2 Socio-Demographic Characteristics of the Research Context

The research context has been the territory of Noci; it has 19,115 inhabitants and has a wide extension (150.60 km^2^), resulting in a low population density (Istat, 01/01/2018) Figure 1. This is an important piece of data: in fact, a population also located in rural areas poses many problems for users and operators for the mutual reachability in the provision of services, in particular for users with a strict health care framework and individuals living alone.

**FIGURE 1:**
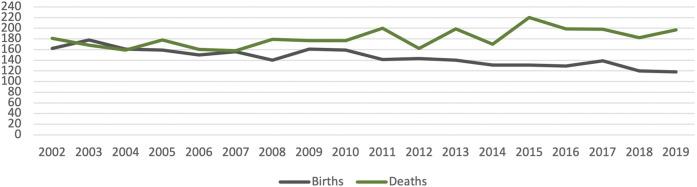
Decreased birth-rate and deaths in Noci. Source: Istat—1st January of each year.

**FIGURE 2:**
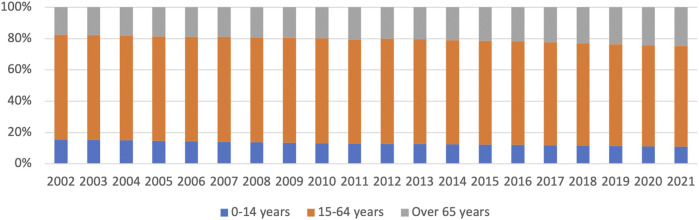
Age structure of the population in Noci (% values)**-** Source: Istat—1st January 2019.

Other factors contribute to this one in the accentuation of the risk of marginality of some categories, for example, the demographic decrease (450 units between 2001 and 2017—Istat data), derive from decreased birth-rate, increased deaths, and braked ingoing migration flow (Figure 2).

All this means that for years, the younger generations have been formed of fewer and fewer members, and this gap is no longer compensated by foreign immigration.

Considering a greater aging of the population (old age index—Istat data: 113,5 in 2002; 197,6 in 2018), it can be easily understood that elderly people (and in any case all those who are not self-sufficient) cannot count on proper human and relational support. This thought is also confirmed by the reduction in the number of members per family [2,86, 2003 vs. 2,51, 2017 (Istat, 1-1-2019)]. Youth forces that could offer more support to people in difficulty are becoming increasingly less, and this results in an increase in the need for institutional support to marginalized groups. From a careful reading of the Area Plan (2016–18)^2^ in the Area of Putignano, there is a general economic impoverishment (about 5.300 inhabitants with less than € 10,000 per year) so that families with greater health and welfare vulnerability are incapable of turning to private structures, if public ones are insufficient or even absent.

Reading and analyzing the Social Zone Plan has been a strategic method for the identification of precise criticalities toward elderly and disabled people:

• Lack of a database and monitoring of their quality of life,
• Lack of family support services,
• Deficiency in the management of Alzheimer’s patients,
• Deficiency in assistance-rehabilitation,
• Lack of specific structures for disabled people,
• Lack of the “After Us,” and
• Deficiency in the resources for home services

In addition to the lack of services in terms of structures and activities/interventions, what is missing is the possibility of data collection, the only one that allows a real opportunity to better intervene. Therefore, the first important step is precisely the commitment to provide resources in order to have a general picture of the situation, since without them, health and welfare intervention would always remain in the emergency and in the contingency. However, the initiative medicine model with the support of digital technology does not only concern users-patients but also operators who will thus have in their hands an instrument as powerful as it is difficult to integrate in their daily historically consolidated practice. For this reason, research has gone far beyond quantitative investigation to explore subjectively perceived needs. To this end, a *focus group* has been set up with various strategic stakeholders in the health and social care sectors.

### 4.3 Focus Group and User Needs

Given the complexity of the research objectives, the technique used for data collection was the focus group. This method of group interview is particularly suitable for promoting and facilitating interaction between participants, contributing in a particular way to the construction of thought (Kitzinger. 1994). Unlike individual interviews that displace people from their social context, focus group discussion creates an important social space of interaction, triggering a higher level of thinking and generating deeper data and insights (Duggleby, 2005; Morgan. 2010). The focus group for exploratory purposes took place on 8 April 2019 at the council hall of the Municipality of Noci. The focus group made it possible to identify three macro-categories of intervention: social, communicative, and technical-operational needs. The study of user representations and expectations was carried out with a specially constructed questionnaire that took into consideration five personal dimensions: sociability, relationships, autonomy, expectations, and user experiences, and highlighted the criticalities and positivity in the dialectic between person and technology.

A type of theoretical-reasoned sampling for representative elements has been used, with the aim of studying strategic stakeholders, welfare and medical workers, and key informers, which can offer a particular abundance of information about the phenomenon in question. There were the following:

• two doctors operating in the Area of Putignano
• a pedagogist
• a psychologist
• a family mediator
• two social workers
• a school home educator
• a technician of the Municipality of Noci
• an integrated home care (nurse)
• a Health Care Partner operator
• The President Retirement Homes “Sgobba” in Noci
• an exponent of the Third Sector and of the volunteer world of the Municipality of Noci.

The composition of the stakeholders is quite heterogeneous. This aspect certainly represented an advantage in a first analysis of the emerging needs, as it helped to draw an interesting multidimensional overview of the needs of the population of Noci. Under the guidance of the conductor/moderator, who, through the guide questions, urged the group to examine in depth the topic of research in a relaxed and non-directional climate, the participants activated meta-reflexive processes on their daily professional practice and on the cultural, social, and geographical context in which they live. The focus group lasted about 130 min and was conducted by an educating sociologist. Based on the research questions and objectives, guide questions were planned with the aim of stimulating conversation while avoiding possible argumentative derailments. The scenario with the guide questions was built following the indications in the literature (Krueger; Zammuner 2013). The meeting was recorded using a voice recorder and faithfully transcribed, despite the difficulties related to the quality of the audio and the characteristics of the council hall of the Municipality of Noci.

The analysis of the scripts was carried out separately by the two interviewers and compared using a qualitative approach of interpretation and subsequent cross-referencing with the intertextual categories that emerged. They were subject to joint analysis, and through this process, it was possible to extract three macro-categories:

• Social Needs
• Communicative Needs
• Technical and Operational Needs

As far as *social needs* are concerned, a widespread educational poverty and the difficulty of emerging social needs have emerged, as it highlights the need to increase the communication of the Municipality of Noci in order to accompany citizens in the complex field of services. Most stakeholders have identified welfare access as the weakest link in the chain and this has an impact on people’s quality of life by increasing their social and health vulnerability. There has often been some resistance from certain sections of the population toward certain social welfare services because of their refusal to seek help. Requests could reinforce discriminatory prejudice to one’s own disadvantage and this feeds self-prejudice and, consequently, self-marginalization. Another element worthy of note is the presence of inexperienced care figures, with particular reference to housemaids, who often do not have adequate basic health education and present difficulties in understanding Italian, which are factors affecting the patients’ quality of life and the correct use of technological devices.

In the field of *communicative needs*, there is an absence of basic information related to the access to welfare and what obviates to the gap between need and performance is often the individuals’ will, for example, a good doctor or a good social worker. Good sector practices, however important they may be, cannot remain in the context of individual discretion, but they must necessarily evolve into efficiency and guarantee of a service. All this has a significant impact on people’s quality of life and this has a domino effect on the whole system. There are many scientific research studies that converge on the importance of the context effect in the treatment and healing processes of the patients. If, on one hand, the research has highlighted the need not to eliminate the already existing database, but to include it according to Cloud modes in greater Cluster, on the other hand, it brings to light a crucial problem linked to a training service turning to operators, families, and caregivers, in order to raise awareness of the available resources and their use.

This is related to *Technical and Operational Needs*. Given the heterogeneous nature of social demand, in relation to the need for *database integration*, the usefulness of interfacing all existing tools has emerged by finding a unique and shared way through *usable Apps*, with simple and intuitive interfaces and a comprehensible language, in order to offer practical diagrams on the operating steps to follow to access the services. The reference to the need to embrace the user-centered design methodology and the ISO 9241-210:2019 legislation during its design is clear, by adopting a design approach of a system that has as its main objective its usability.

Even if ITCs open up scenarios of effective equality and social inclusion, as well as individual and collective empowerment, they can represent, especially in culturally and economically marginal areas, a new form of discrimination by increasing the digital divide: the number of people who do not know how to use IT applications has grown considerably, especially those supporting public service and welfare access.

This is due to the different language and logic between those who produce technology and those who use it. For these reasons, the reference to a really user-centered approach, known as *UCD, User-Centered Design*, described in the previous paragraphs, could avoid the risk of negative effects.

#### 4.3.1 Swot Analysis

In relation to what emerged from the demographic factors, from the reading of the Area Plan and from the elements detected by the subsequent qualitative survey through needs analysis through focus groups, it was possible to develop a Swot matrix:

**Table:** 

	Benefits/opportunities	Risks/dangers
Internal	Massive presence of Third Sector services	Increase in the senility of the population
	Good industry practices	Decrease in births
	Willingness to improve the offer of services	Decrease in number and composition of households
		Widespread cultural and educational poverty
		Little-known access welfare
External	Collaboration between the municipal institution and regional IT companies for the use of hardware and software technology in the smart social field	Operator know-how often inadequate to technological changes
	Synergy between humanistic and engineering research	Failure to share a common culture of intervention

### 4.4 From the User’s Needs to the Redefinition of the Technological Proposal. The Pedagogical Perspective

The involvement of the end-users has been fundamental to collecting qualitative and quantitative data on the effectiveness of the product and to ensure a design for all.

In order for telemedicine initiatives associated with the needs of fragile elderly people to produce satisfactory results, it was necessary to take a multidisciplinary approach, in which the synergy between medical figures, producers/designers, and pedagogists could be functional to the real improvement of the person’s well-being. The assumption of bio-psycho-social perspective (World Health Organization Europe, 2006) and the constructs of quality of life and participation pave the way for inclusive logics which implement a deep and questioning reflection on all contexts of life, with the aim of exposing the set of disabling processes and indicating a valid ally in the use of technological resources. In the psycho-pedagogical area, also because of the diagnostic classification of the ICF, we can identify a very interesting line of research whose focus concerns the quality of life of the person with disadvantages and disability; the models and tools for identifying needs in view of aging; the inclusion and care of the fragile elderly person; the risks to social isolation, and, last but not least, the changes in the caregivers’ networks that the new social structures have determined (Pinnelli, 2014, pp. 1–13).

Moving from that prospective was create and used an explanatory questionnaire, with the aim to resize some starting hypotheses and work packages of the D-Sys-Com project with regard to an adaptation of the design of the products in relation to the users. The focus of the questionnaire has been to explore the relationship between the elderly person and technology; then, using the special pedagogy has the epistemological basis of the work.

As suggested by Pinnelli (cit.), the idea behind the detection tool used to investigate some characteristics of end users so that a real participatory design process can be realized that can effectively contribute to a better definition of systems and tools, regardless of the technologies used. The relevance to the need to embrace the *user-centered design methodology* during the design and testing phase, adopting an approach to the design and testing of a system that has as its main objective its usability.^3^ The lack of inclusive architecture in medical sensors and technologies related to telemedicine not only constitutes a predictor of disability but also creates it, as the ICF suggests: “it is the environment where the person lives that determines and concretizes the disability” (Bickenbach et al., 2008).

#### 4.4.1 The Questionnaire: Perceptions, Expectations, and Preferences Toward ICT

Precisely by virtue of going in the direction discussed above, the CNTHI researchers of the University of Salento have prepared an analysis tool that has taken into consideration various spheres of the person. Sociality, relationships, autonomy, expectations, and experiences of use represented compasses to probe the usability of complex tools, with the aim of achieving a true process of media education considered part of a wider form of democratic citizenship (Buckingham, 2006, p. 32).

The tool developed in this study consists of 23 questions divided into 5 sections: 1) demographic characteristics; 2) technology experience; 3) perception of technology; 4) personal expectations regarding their quality of life and technology; and 5) characteristics of technologies considered preferential.


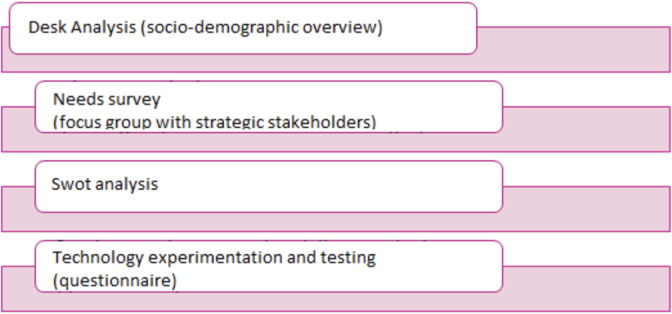


Due to the restrictions linked to the anti-contagion measures following the Covid-19 pandemic, it was not possible to administer the questionnaires in the presence of the CNTHI researchers, but the social worker of the Municipality of Noci was embroiled in order to guarantee a connecting link with the elderly people involved.

#### 4.4.2 Results. General Information on the Cross-Section

The cross-section includes 18 patients living within the municipal territory of Noci, 11 women and 7 men. The age is quite high. The average age is in line with the life expectancy threshold in Italy, measured for the year 2018 by ISTAT: 80.8 years for men and 85.2 for women; in particular, female patients have an average life expectancy slightly lower than Italian women (83.7), while for men it is almost the same (85.5). At least half of the subjects in the cross-section are 83 years old up to a maximum declared of 100, while among the “youngest,” they reach a minimum age of 71.

It is worthy to highlight that age was not a primary criterion for the selection of the cross-section, because the psychological and physical status of the subject involved and the availability of the main caregiver were a priority. During the phase of identification of the cross-section, thanks to the availability of some general practitioners of the Municipality of Noci, patients with a variety of diseases were pointed out: people with cognitive impairment (11); in four cases, the condition was not accompanied by other pathologies, while in two cases, it was accompanied by COPD, one of which even by chronic renal insufficiency, others with kidney or respiratory or heart failure. Among the most common cases, there were also people with diabetes and heart problems (4), almost always in conjunction with other morbidity; finally, there were three cases with respiratory failure Table 1.

**TABLE 1:** Synoptic picture of the declared morbidity.

Cognitive impairment (tot. 11)	Chron. obstr. pulmon. disease	2 (1 witd chronic kidney failure)
	Polypatdologies	2
	Resp. insuff.	1
	Kidney insuff.	1
	Heart failure	1
Diabetes (tot. 4)		
	Neurovasc. complic.	1
	Heart failure	2
	Visually impaired	1
Respiratory insufficiency (tot. 2)	Heart disease	1
	Heart failure	1
Paraparesis (tot.1)		1

#### 4.4.3 Demographic Characteristics

The elderly people to whom the questionnaire was administered are predominantly widowers/widows (55.5%); 22.2% were single, and the remaining 22.2% declared themselves to be married and living with their spouse.

The level of education held by the cross-section is almost entirely relative to primary school (90%), followed by a 10% who declare not to possess a level of education. On average, the duration of the study path of the interviewed cross-section is 5 years.

The working position of the cross-section is distributed as follows: housewife (56.25%), cook (12.5%), farmer (12.5%), auxiliary (6.5%), and tailor (6.5%).

In most cases, respondents declare that they live alone (44.44%), while those who live with their spouse constitute 22.22% of our cross-section; in the same way, those who live with a housemaid or their children constitute 16.66% of the respondents.

More than half of the cross-section live in a flat (66.6%) and the remaining 33.33% in detached houses.

#### 4.4.4 Experience With Technology

**FIGURE 3:**
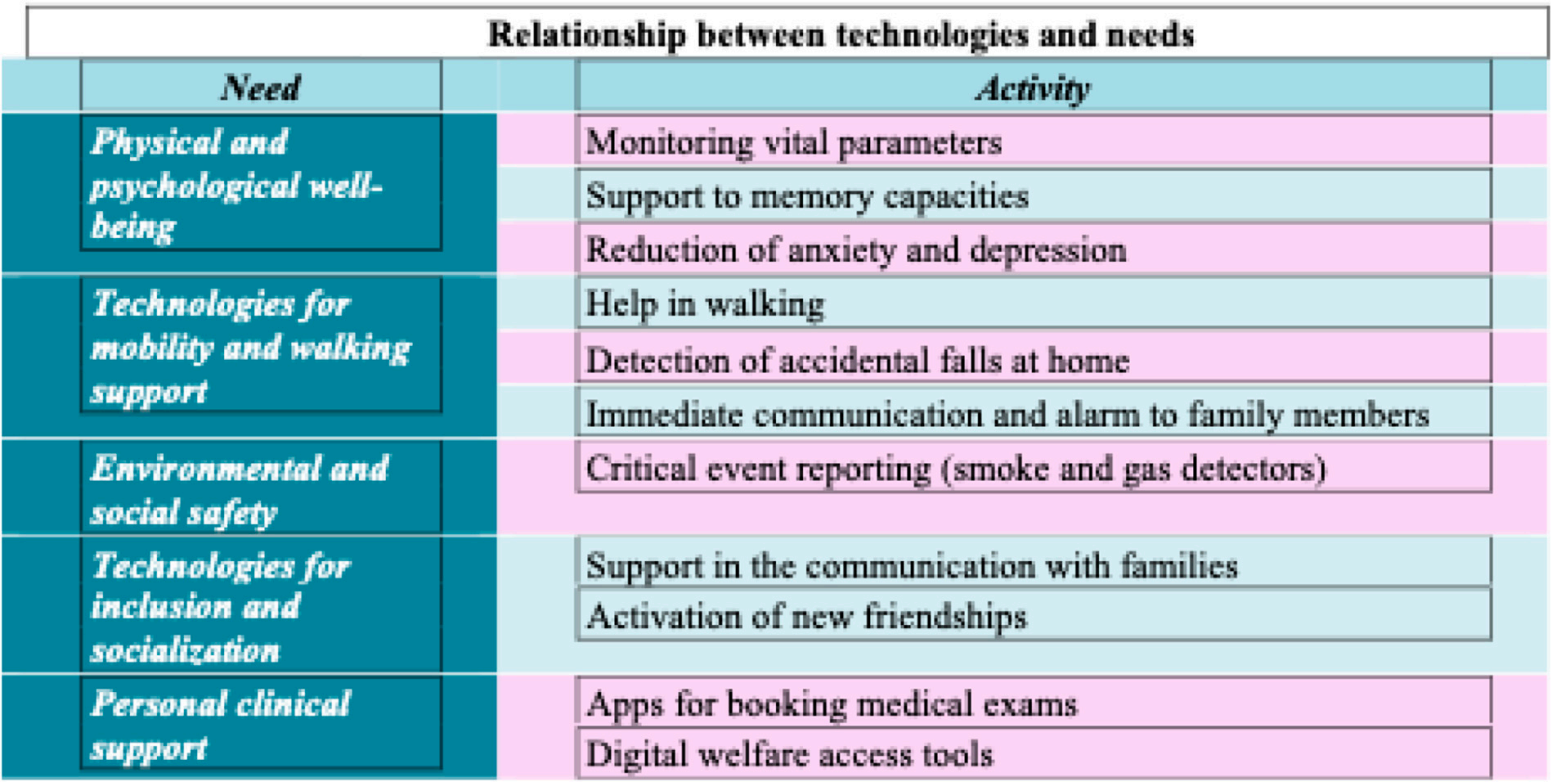
Relationship between technologies and needs.

Respondents attributed a value of importance on a 5-point Likert scale to technologies that could meet their needs. The following Figure 3 summarizes the sector of the needs that technologies should meet.

**FIGURE 4:**
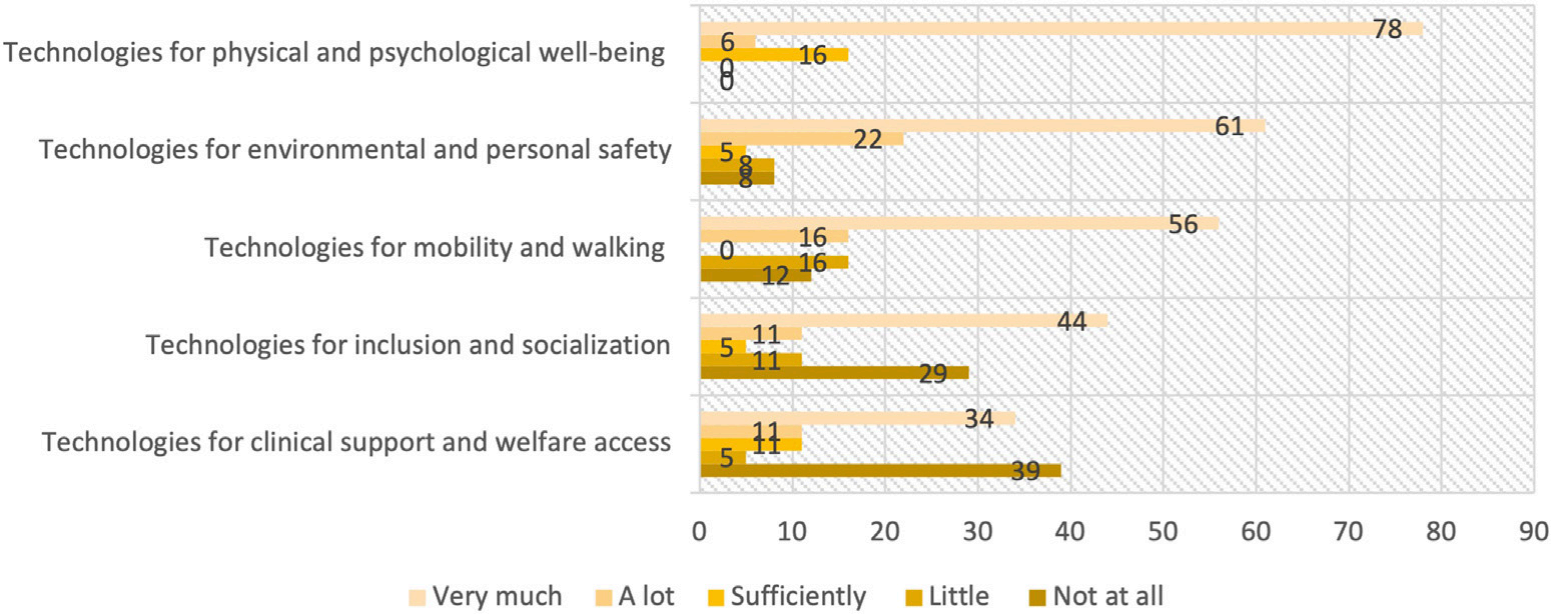
Technology relevance and sectors.

The following order shows the percentages expressed by our reference cross-section. The order is by priority, also presented in Figure 4.

Technology relevance and sectors.

1) Technologies for physical and psychological well-being
2) Technologies for environmental and personal safety
3) Technologies for mobility and walking support
4) Technologies for inclusion and socialization
5) Technologies for clinical support and welfare access

As regard the **experience** that users have gained in relation to the technologies possessed during their life, the questionnaire found a very limited use of technological tools: 94.44% of respondents, that is, almost all the cross-section, declare that they use landlines, while mobile phones are used by 66.66%, followed by 11.11% who claim to have wi-fi at home. The same percentage can be found for those who use fixed sensors for the detection of smoke and gas leaks (11.11%), and, finally, only 5.55% is equipped with an alarm system. Nobody owns medical alert bracelets, video intercom, wi-fi switches for shutters, smart thermostats, automatic lights, laptops, or personal computers.

**FIGURE 5:**
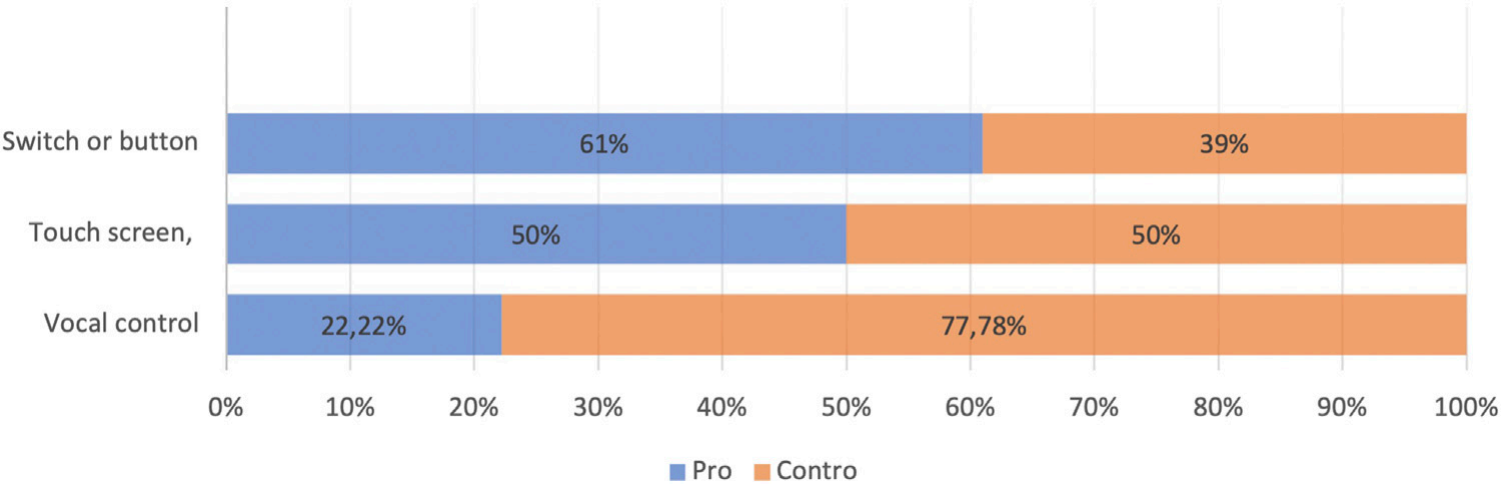
Command mode preferences.

About the degree of **satisfaction related to the technological tools used in daily life**, we point out the following degree of importance that users attribute to their devices: landline was attributed a great deal of importance (62.2%), followed by 12.5% who give it a lot of importance and 25% who give it sufficient importance. Mobile phone was given a great deal of importance (52.25%), followed by 35% of the cross-section who gave no importance and 12% who gave a lot of importance (Figure 5).

The impact of the **socio-cultural and demographic** sphere can also be considered within the judgement given to other technologies: the possession of a digital infrastructure such as Internet is not considered important at all by about 87% of the cross-section, followed by 13% who give it great importance. We can find similar positions also for the use of computers, desktops, or laptops: not important at all (79%) and little importance (21%). Similar percentages are detectable in other devices: wearable sensors were given much importance by 14% of the cross-section compared to 86% who give it little or no importance. Finally, both smoke sensors and alarm systems were attributed a lot of importance by 7% of the cross-section compared to 92% who had little or no interest at all in them.

**FIGURE 6:**
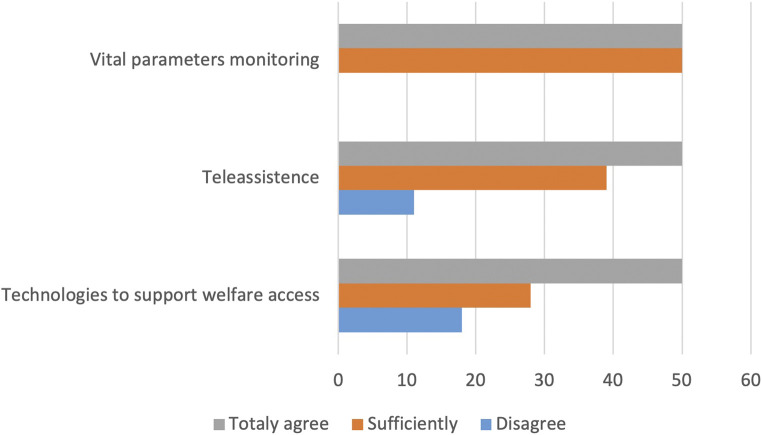
Perception of effectiveness.

In relation to the **use of technological** devices to be tested, the questionnaire observed useful advice (Figure 6) in order to ensure usable and effective systems, able to positively affect the quality of life of the subjects involved without distorting their behavior. The respondents declared that for a device that improves their quality of life, the control mode they prefer is a remote control with buttons to press (61%), followed by touch screen (50%). Vocal control is less welcome (22.22%).

#### 4.4.5 Users’ Expectations on Innovative and Technological Tools. Perception of Effectiveness

With regard to the **overall improvement in the quality of life** through the use of technologies, 44.44% of the cross-section attributed a great deal of importance to their use, 38.88% declared that they have a sufficient impact, while 11.13% claim not at all and 5.55% a little.

**Expectations regarding technologies** with specific reference to some aspects of the quality of life are the following:

- maintenance/monitoring of physiological parameters and personal mobility represent area where expectations toward technologies are very high (55.55%):
- technologies to support psychological well-being (51%),
- technologies to support daily routine and to increase personal autonomy (44%), and, finally,
- technologies and devices useful in the management of living spaces and household (38,88%).

In relation to the results of the questionnaire and the previous phases of the research (focus groups and in-depth interviews with strategic stakeholders), very significant data emerged with reference to the degree of effectiveness perceived by the sensors’ object of experimentation. There are three domains chosen to evaluate the perception of effectiveness of Smart Social technologies (Figure 6): vital parameters, teleassistance, and technologies to support welfare access (booking medical exams online, apps to facilitate service access, and orientation).

From reading the graph, elderly people express their opinion of total agreement in asserting the perception of effectiveness of tools used to monitor the vital parameters at a distance (50%), while the other half of the cross-section seems quite in agreement. Regarding teleassistance instruments aimed at reducing isolation and social and health vulnerability, the assessment is as follows: totally agree (50%), sufficiently agree (39%), and disagree (11%). Finally, with regard to technology aimed at facilitating welfare access and accompanying citizens-users in the vast area of services, the perception of effectiveness of these devices registers as follows: totally agree (50%), sufficiently agree (28%), and disagree (16%).

The survey linked to the perception of the effectiveness of technological tools has deepened the importance that respondents assign to certain **requirements**. According to the cross-section, the most important features of a technological tool that improves their quality of life are usefulness (94%), followed by simplicity and safety (83%). They are followed by little physical strength in order to use them (50%), sustainability of costs (27.77%), and invasiveness (22.22%) at the same level as aspects related to privacy and protection of sensitive data (22.22%).

## 5 Discussion

As regards the processes of technological design, one of the most significant concepts is about atomic design exposed in the homonymous book by Bread Froast (2016), for a much more holistic design approach, in a perspective in which the components of design (atom, molecule, organism, and product) synergically coexist and the design of each of them has concentric effects on the whole society (cit.). It is necessary to combine every single atom of design with the person’s needs. As a means of social transformation, of inclusion, of broadening the range of opportunities for participation, and of improving the quality of life, technology has a strong political character. Papanek (1985), in his book *Design for the real world*, stated that products very often leave behind the real problems of people by placing themselves as the tip of an iceberg, under which excluding logic lies. This occurs whenever you plan in a non-deliberate manner. In the design of complex systems, it is necessary to act in the best possible way, in what is known as the atomic design of technologies, because creating excluding products will create excluding societies (1985). As suggested by Pinnelli (2014), international research highlights the cruxes of the digital divide for elderly people, highlighting how much it is influenced not only by age but by a whole set of demographic, cultural, and experiential factors. The study aimed to deepen the knowledge of the end-users for whom the development project of telemedicine services is intended; what emerged from the swat analysis of the services and documents referring to the Area Plan and from the focus group with the stakeholders helped to calibrate the exploration areas of the questionnaire. In line with the literature, it was decided to deepen the exploration of the psychosocial factors of the users: attitudes, expectations, degree of satisfaction, and trust toward telemedicine services.

In relation to the needs mostly felt by respondent patients, technologies should fulfill functions related to the protection and maintenance of physical and psychological well-being, followed by mobility support, environmental and personal safety, clinical support, and, last in order of preference, socialization and social inclusion.

The data on the possession of Internet at home is very important and should be considered for testing and design medical sensors and telemedicine and welfare tools, as the unavailability of a digital infrastructure for users should make designers and partner companies aware of the need to find appropriate solutions.

The lack of importance attributed to mobile phones is considerable and can be traced back to the socio-demographic conditions of our cross-section made up of a fairly high average age and levels of education attested to primary school. This data is also confirmed in the literature that attests to difficulties in using the mobile phone to self-monitor the health problems of frail and elderly patients “Patients voiced concerns about lack of knowledge or experience taking photos with a cellphone and potentially dysfunctional factors…his or her ability to properly visualize the wound-area or uncertainty about their “state-of-mind” after surgery (Wiseman, J. T., et alii. 2015).

The second domain of the questionnaire relates to users’ expectations of innovative and technological tools. In the previous exploratory phase of the research (stakeholder focus group, 8 April 2019) the heterogeneous nature of the social demand needed to identify a single and shared path through the creation of a usable tool, with a simple and intuitive interface and an understandable language. The *user-centered design* requires an approach to the design of a system that has as its main objective its usability (Vandi and Nicoletti, 2013). From the previous focus group and subsequent in-depth interviews with some strategic stakeholders, the main problem with telemedicine and teleassistance tools was mainly related to the distrust in technology, a cause of complications and stress in the specific context of daily use of the user.

- D-Sys-Com should support the needs of physical and psychological well-being, followed by the need to ensure environmental and personal safety, the need for mobility and walking, followed by the need to ensure and support social inclusion, and, finally, clinical support and access to welfare by implementing functions, services, and applications that meet the related needs.
- D-Sys-Com experimenting and testing technologies must be designed in accordance with the criteria of usefulness, simplicity, and safety, followed by the identification of minimally invasive devices that do not require great physical strength for their use. They are followed by the importance linked to cost sustainability and respect for user’s privacy. For 22% of users, privacy constitutes a problem to deal with, a percentage that is not high compared to other literature data (ibidem) but which is correlated with the level of education of users.
- The answers attest preference for solutions that can be managed by users through a simple remote control with buttons to press.
- Given the poor experience of using technological devices by the cross-section, the adoption of highly intuitive solutions that do not require the modification of user behavior in their specific daily context is suggested. Considering these conclusions, technologies can become exceptional tools in order to improve people’s quality of life and, depending on how they are designed, implemented, and applied, can act as facilitators or obstacles/barriers in the performance of the usual activities, placing themselves in the gap indicated by the ICF of the WHO between capacity and performance, that is, what the person can do based on his functional residue and what the person can do with the support of environmental mediators. While as regards the first one, there is a little leeway, with elderly people, it is clear that we can act to the advantage of the latter.

A particularly important aspect is the cultural level of users; it is a determining factor in the acceptance of technologies, in digital literacy, and in the ability to appreciate their use. The data emerged about the privileged interaction methods (remote control), the expectation in terms of security, and the great importance attributed to the fixed network attest to a strong anchoring to a limited perception of technological support, although in many questions, promising trust is glimpsed toward innovation.

These reflections lead us to agree with the idea that the digitalization of public health should integrate the following pillars: political commitment, regulatory frameworks, technical infrastructures, targeted economic investments, education, research, monitoring, and evaluation (Odone et al., 2019).

In the field of telemedicine and teleassistance, inclusiveness should not be a goal, but a methodology: this means that people are not considered on the basis of congenital characteristics, but in their entirety. This completely turns a paradigm upside down and necessarily stimulates two questions: what does it mean to consider inclusivity as a methodology and not as a goal? And what does inclusivity really mean? Inclusion requires a holistic approach to design and necessarily requires one to embrace a cross-disciplinary approach, adapting to the needs of people what you design. To include means to consider the person in the individuality and not grouped in view of the person in his/her individuality, not labels or clusters. This means defining customized adaptations and interventions. The process implemented in the research described had the purpose of reorienting the development of the service envisaged by the D-Sys-Com project and the questionnaire defined turned out to be a good exploratory tool which, together with the other sources of analysis, made it possible to highlight the trajectory development that the remote assistance system should have adopted.

## Data Availability

The raw data supporting the conclusion of this article will be made available by the authors, without undue reservation.

## Footnotes

1 A project developed for the participation to the PO.FESR-FSE 2014–20 calls, measure IV “Electronic government for Elderly People.” The project partners are 3 municipalities within the regional territory of Apulia and hardware and software producers with the scientific support of the Interdisciplinary Laboratory of Design and Integrated Management of Industrial Plants of the University of Salento. The project is divided into 3 domains: “Smart Building” belonging to the Municipality of Nardò (LE), “Smart City” within the Municipality of Conversano (BA), and, finally, “Smart Social” within the Municipality of Noci.

2 The Zone Plan is the territorial planning tool of the Municipalities for the development of a local policy of services and social interventions.

3 Usability is meant as “the ability of a product to facilitate a specific user in achieving specific objectives with effectiveness, efficiency and satisfaction in a specific context of use.”
